# Impact of body mass index on surgical outcomes of gastric cancer

**DOI:** 10.1186/s12885-018-4063-9

**Published:** 2018-02-06

**Authors:** Fan Feng, Gaozan Zheng, Xiaohua Guo, Zhen Liu, Guanghui Xu, Fei Wang, Qiao Wang, Man Guo, Xiao Lian, Hongwei Zhang

**Affiliations:** 1 Division of Digestive Surgery, Xijing Hospital of Digestive Diseases, Fourth Military Medical University, 127 West Changle Road, Xi’an, Shaanxi 710032 China; 2 Department of General Surgery, No. 534 Hospital of PLA, West Lichun Road, Luoyang, Henan 471000 China; 3 Department of General Surgery, No. 91 Hospital of PLA, 239 Gongye Road, Jiaozuo, Henan 454000 China

**Keywords:** Gastric cancer, Complications, Prognosis, BMI

## Abstract

**Background:**

The association between body mass index (BMI) and clinical outcomes of gastric cancer were still under debate. The aim of the present study was to investigate the impact of BMI on intraoperative conditions, postoperative complications and prognosis of gastric cancer.

**Methods:**

From October 2008 to March 2015, 1210 gastric cancer patients treated with D2 gastrectomy were enrolled in the present study. Patients were divided into three groups: low BMI group (BMI < 18.5 Kg/m^2^), normal BMI group (18.5 Kg/m^2^ ≤ BMI < 25.0 Kg/m^2^) and high BMI group (BMI ≥ 25.0 Kg/m^2^). Clinicopathological characteristics and prognosis of patients were recorded and analyzed. Propensity score matching was used to match patients in the three groups.

**Results:**

There were 107 patients in low BMI group (8.9%), 862 patients in normal BMI group (71.2%) and 241 patients in high BMI group (19.95%). Before matching, BMI was inversely associated with tumor size, tumor depth, lymph node metastasis (LNM) and tumor stage (all *P* < 0.05). After matching, the clinicopathological features were all comparable among the three groups (all *P* > 0.05). High BMI was associated with increased blood loss and operation time, and deceased number of retrieved lymph nodes (all *P* < 0.05). For postoperative complications, low BMI was associated with decreased rate of postoperative fever (*P* = 0.025). Age, BMI, tumor size, Borrmann type, pathological type, type of gastrectomy, tumor depth, LNM and tumor stage were risk factors for the prognosis of gastric cancer. Multivariate analysis showed that only BMI, tumor size, tumor depth and LNM were independent prognostic factors. The overall survival of patients with low BMI was significantly worse than patients with normal (*P* < 0.05) or high BMI (*P* < 0.05). However, the overall survival was comparable between patients with normal and high BMI (*P* > 0.05).

**Conclusions:**

BMI was inversely associated with tumor size, tumor depth, LNM and tumor stage. High BMI was associated with increased blood loss and operation time, and deceased number of retrieved lymph nodes. Low BMI was associated with decreased rate of postoperative fever and decreased survival.

## Background

Gastric cancer is the fourth most common cancer in the world [1] and the second most common cancer in China [2]. Surgical resection with extended lymph node clearance remains the only curative treatment for gastric cancer. It is often diagnosed at an advanced stage. Thus, the prognosis of gastric cancer is still not promising, even with the rapid advances in surgical techniques and adjuvant therapy [3].

Tumor patients always tend to suffer from malnutrition and lose weight because of decreased oral intake and alterations in metabolism [4]. The incidence of malnutrition in tumor patients is reported to be ranged from 10% to 85% according to the type, location and stage, etc. [4]. Body mass index (BMI) was an effective measurement for evaluating nutritional status of cancer patients [5]. In recent years, the associations between BMI and clinical outcomes of cancer patients have been widely investigated [6–9], including gastric cancer [10]. Some studies reported that BMI was associated with postoperative complications [4] and prognosis [11] of gastric cancer. However, no association between BMI and clinical outcomes of gastric cancer has also been reported [12, 13].

Given this situation, the aim of the present study was to investigate the impact of BMI on the clinical outcomes of gastric cancer.

## Methods

This study was performed in the Xijing Hospital of Digestive Diseases affiliated to the Fourth Military Medical University. From October 2008 to March 2015, 1210 gastric cancer patients treated with D2 gastrectomy were enrolled in the present study. All patients were treated with total, proximal or distal D2 gastrectomy. The surgical procedure was based on the recommendations of the Japanese Gastric Cancer Treatment Guidelines [14]. The postoperative chemotherapy was given to patients according to the NCCN guideline for gastric cancer. This study was approved by the Ethics Committee of Xijing Hospital, and written informed consent was obtained from all patients before surgery.

Clinicopathological data including gender, age, BMI, tumor location, tumor size, Borrmann type, pathological type, type of gastrectomy, tumor depth, lymph node metastasis and tumor stage were collected. Surgery-related data including blood loss, operation time, number of retrieved lymph nodes and length of postoperative stay were recoded. Postoperative complications within 30 days including pneumonia, fever, wound disruption, wound infection, abdominal bleeding, anastomosis leakage, chyle leakage, gastric stasis, pleural effusion and ileus were also recorded through telephone and outpatient follow up. The survival of patients was followed up till November 2016 every 3 months.

BMI was calculated as body weight (kilograms) divided by height (meters) squared. Patients were divided into three groups according to BMI level: low BMI group (BMI < 18.5 Kg/m^2^), normal BMI group (18.5 Kg/m^2^ ≤ BMI < 25.0 Kg/m^2^) and high BMI group (BMI ≥ 25.0 Kg/m^2^).

To reduce bias, propensity score matching was used in our present study. The parameters used for propensity score matching was age, gender, tumor location, tumor size, type of resection, pathological type, tumor depth and LNM.

Data were processed using SPSS 22.0 for Windows (SPSS Inc., Chicago, IL, USA). Discrete variables were analyzed using Chi-square test or Fisher’s exact test. Continuous variables were expressed as median (interquartile range) and analyzed using nonparametric test. Significant prognostic risk factors identified by univariate analysis were further assessed by multivariate analysis using the Cox’s proportional hazards regression model. Overall survival was analyzed by Kaplan-Meier method. The *P* value was considered to be statistically significant at 5% level.

## Results

There were 949 male (78.4%) and 261 female (21.6%). The median age was 59 years (20–87). There were 107 patients in the low BMI group (8.9%), 862 patients in the normal BMI group (71.2%) and 241 patients in the high BMI group (19.95%). The median follow-up of the low, normal and high BMI group was 22.4 (1.3–66.2) months, 25.0 (1.4–73.5) months and 25.0 (1.6–74.6) months, respectively. The associations between clinicopathological characteristics and BMI were summarized in Table 1. The results showed that BMI was not associated with age, gender, tumor location, Borrmann type, differentiation status and type of resection (all *P* > 0.05). However, BMI was inversely associated with tumor size, tumor depth, LNM and tumor stage (all *P* < 0.05).

**Table 1:** Correlation between clinicopathological characteristics and BMI before matching

Characteristics	Low BMI (*n* = 107)	Normal BMI (*n* = 862)	High BMI (*n* = 241)	*P* value
Gender				
Male	83	677	189	0.974
Female	24	185	52	
Age				
≤ 60	66	501	134	0.558
> 60	41	361	107	
Tumor location				
Upper third	33	300	92	0.327
Middle third	15	155	31	
Lower third	53	349	100	
Entire	6	58	18	
Tumor size (cm)				
≤ 5	61	574	175	0.016
> 5	46	288	66	
Borrmann type				
I	19	102	34	0.498
II	29	232	59	
III	37	305	84	
IV	12	64	13	
Pathological type				
Well differentiated	6	74	24	0.511
Moderately differentiated	22	218	68	
Poorly differentiated	74	540	140	
Signet ring cell or Mucinous	5	30	9	
Type of gastrectomy				
Proximal	7	78	25	0.594
Distal	41	316	97	
Total	59	468	119	
Tumor depth				
T1	10	161	52	0.047
T2	10	71	30	
T3	39	308	86	
T4a	46	315	72	
T4b	2	7	1	
Lymph node metastasis				
N0	23	291	99	0.005
N1	20	141	49	
N2	20	153	33	
N3a	26	182	45	
N3b	18	95	15	
Tumor stage				
Ia	8	140	47	0.006
Ib	3	50	17	
IIa	10	100	34	
IIb	19	122	45	
IIIa	21	109	27	
IIIb	16	163	37	
IIIc	30	178	34	

To reduce bias, propensity score matching was used to match patients in the three groups. After matching, there were 104 patients in the low BMI group, 416 patients in the normal BMI group and 104 patients in the high BMI group. The clinicopathological features were comparable among the three groups after matching (Table 2, all *P* > 0.05).

**Table 2:** Correlation between clinicopathological characteristics and BMI after matching

Characteristics	Low BMI (*n* = 104)	Normal BMI (*n* = 416)	High BMI (*n* = 104)	*P* value
Gender				
Male	82	331	76	0.353
Female	22	85	28	
Age				
≤ 60	60	220	62	0.379
> 60	44	196	42	
Tumor location				
Upper third	33	156	37	0.329
Middle third	15	62	13	
Lower third	51	160	41	
Entire	5	38	13	
Tumor size (cm)				
≤ 5	60	251	60	0.818
> 5	44	165	44	
Borrmann type				
I	19	59	11	0.682
II	27	110	32	
III	37	162	42	
IV	11	35	7	
Pathological type				
Well differentiated	6	23	4	0.746
Moderately differentiated	21	94	29	
Poorly differentiated	72	284	65	
Signet ring cell or Mucinous	5	15	6	
Type of gastrectomy				
Proximal	7	36	6	0.816
Distal	41	148	38	
Total	56	232	60	
Tumor depth				
T1	10	50	12	0.721
T2	10	35	14	
T3	39	143	40	
T4a	44	184	38	
T4b	1	4	0	
Lymph node metastasis				
N0	23	105	22	0.730
N1	20	61	19	
N2	20	87	16	
N3a	25	105	33	
N3b	16	58	14	
Tumor stage				
Ia	8	40	10	0.280
Ib	3	24	6	
IIa	10	39	9	
IIb	19	59	16	
IIIa	21	41	12	
IIIb	16	109	26	
IIIc	27	104	25	

The association between BMI and surgery-related parameters were shown in Table 3. The results showed that patients in the high BMI group was associated with increased blood loss and operation time, and deceased number of retrieved lymph nodes (all *P* < 0.05). The length of postoperative stay was comparable among the three groups (*P* = 0.179).

**Table 3:** Comparison of surgery-related parameters after matching

Characteristics	Low BMI	Normal BMI	High BMI	*P* value
Blood loss (ml)	150 (100, 200)	150 (100, 200)	200 (150, 350)	< 0.001
Operation time (min)	170 (140, 220)	185 (150, 230)	217.5 (175, 263.75)	< 0.001
Number of retrieved lymph nodes	26 (22, 33)	26 (21, 32)	23 (19, 27)	< 0.001
Length of postoperative stay	7 (6, 9)	7 (6, 9)	8 (6, 9)	0.179

The association between BMI and postoperative complications were shown in Table 4. The results showed that patients in the low BMI group was associated with decreased rate of postoperative fever (*P* = 0.025). However, BMI was not associated with other complications (all *P* > 0.05).

**Table 4:** Comparison of postoperative complications after matching

Complications	Low BMI	Normal BMI	High BMI	*P* value
Fever	8	74	21	0.025
Pneumonia	6	32	8	0.788
Wound infection	0	1	0	0.778
Wound disruption	0	4	2	0.364
Anastomosis leakage	0	9	0	0.102
Abdominal bleeding	1	4	0	0.604
Chyle leakage	2	5	0	0.405
Pleural effusion	1	11	1	0.382
Gastric stasis	0	0	1	0.082
Ileus	1	8	2	0.794

The risk factors for the prognosis of gastric cancer patients were analyzed using univariate analysis and shown in Table 5. The results showed that age, BMI, tumor size, Borrmann type, pathological type, type of gastrectomy, tumor depth, LNM and tumor stage were associated with the prognosis of gastric cancer. Multivariate analysis showed that only BMI, tumor size, tumor depth and LNM were independent prognostic factors (Table 6).

**Table 5:** Univariate analysis of risk factors for prognosis of gastric cancer after matching

Prognostic factors	β	Hazard ratio (95% CI)	*P* value
Gender	−0.127	0.881(0.643–1.206)	0.428
Age	0.326	1.386(1.075–1.786)	0.012
BMI	−0.256	0.774(0.619–0.969)	0.025
Tumor location	0.056	1.058(0.933–1.199)	0.380
Tumor size	0.769	2.158(1.670–2.787)	< 0.001
Borrmann type	0.357	1.429(1.260–1.621)	< 0.001
Pathological type	0.467	1.596(1.289–1.975)	< 0.001
Type of gastrectomy	0.432	0.649(0.522–0.808)	< 0.001
Tumor depth	0.854	2.348(1.929–2.858)	< 0.001
Lymph node metastasis	0.591	1.807(1.586–2.058)	< 0.001
Tumor stage	1.239	3.451(2.575–4.623)	< 0.001

**Table 6:** Multivariate analysis of risk factors for prognosis of gastric cancer after matching

Prognostic factors	β	Hazard ratio (95% CI)	*P* value
Age	0.198	1.219(0.940–1.582)	0.136
BMI	0.332	0.717(0.570–0.903)	0.005
Tumor size	0.345	1.412(1.077–1.851)	0.013
Borrmann type	0.080	1.083(0.943–1.244)	0.259
Pathological type	0.120	1.128(0.900–1.414)	0.297
Type of gastrectomy	−0.065	0.937(0.743–1.181)	0.582
Tumor depth	0.560	1.751(1.397–2.193)	< 0.001
Lymph node metastasis	0.403	1.496(1.298–1.724)	< 0.001

**Fig. 1:**
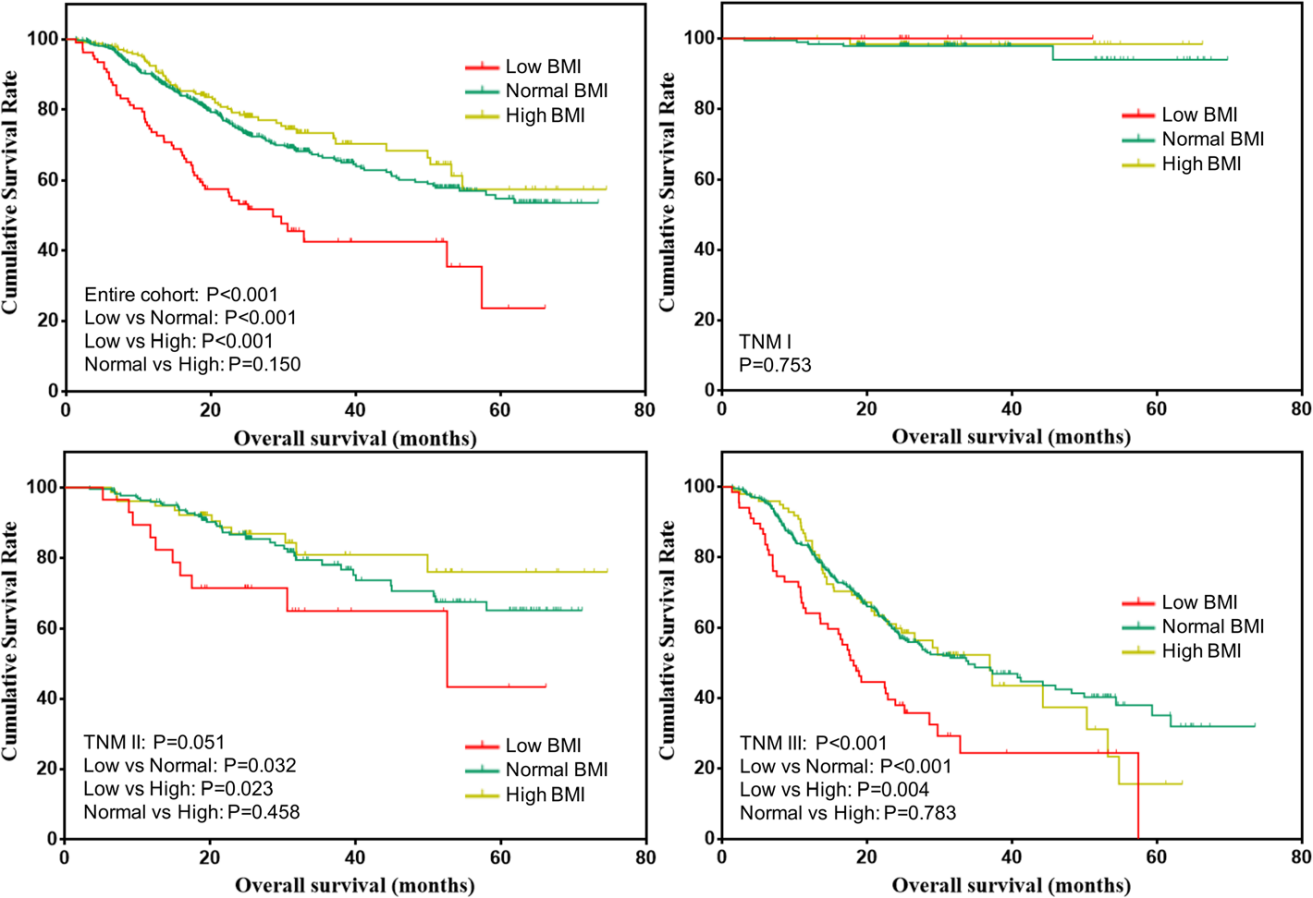
Overall survival of gastric cancer patients stratified by BMI before matching

The overall survival of gastric cancer patients stratified by BMI was shown in Fig. 1. The overall survival of patients with low BMI was significantly worse than patients with normal (*P* < 0.001) or high BMI (*P* < 0.001). However, the overall survival was comparable between patients with normal and high BMI (*P* = 0.150). Further, the overall survival of patients stratified by tumor stage were analyzed. For stage I patients, the overall survival was comparable among the three groups (*P* = 0.753). For stage II patients, the overall survival of patients with low BMI was significantly worse than that with normal (*P* = 0.032) or high BMI (*P* = 0.023). The overall survival of patients with normal and high BMI was comparable (*P* = 0.458). For stage III patients, the overall survival of patients with low BMI was significantly worse than that with normal (*P* < 0.001) or high BMI (*P* = 0.004). The overall survival of patients with normal and high BMI was comparable (*P* = 0.783).

**Fig. 2:**
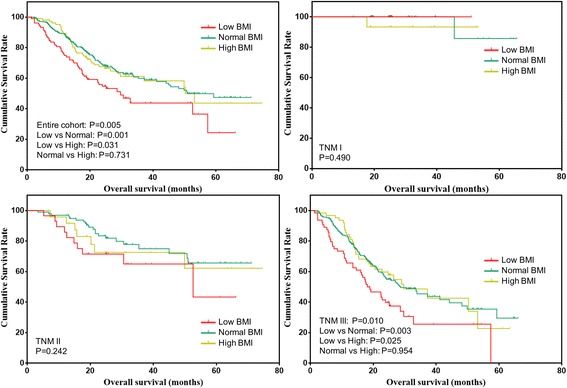
Overall survival of gastric cancer patients stratified by BMI after matching

The overall survival was also analyzed for patients after propensity score matching (Fig. 2). The overall survival of patients with low BMI was significantly worse than patients with normal (*P* = 0.001) or high BMI (*P* = 0.031). However, the overall survival was comparable between patients with normal and high BMI (*P* = 0.731). Further, the overall survival of patients stratified by tumor stage were analyzed. For stage I and II patients, the overall survival was comparable among the three groups (both *P* > 0.05). For stage III patients, the overall survival of patients with low BMI was significantly worse than that with normal (*P* = 0.003) or high BMI (*P* = 0.025). The overall survival of patients with normal and high BMI was comparable (*P* = 0.954).

## Discussion

BMI is a widely used parameter in clinical practice due to easy measurement. BMI is associated with a variety of cancer, including gastric cancer [15]. A meta-analysis has demonstrated that high BMI was associated with increased risk of gastric cardia cancer [16]. The association between BMI and clinical outcomes of gastric cancer has also been widely investigated, however, it was still under debate [10–13]. Thus, the present study aims to investigate the impact of BMI on the clinical outcomes of gastric cancer after radical gastrectomy. We found that BMI was inversely associated with tumor stage. High BMI group was associated with increased blood loss and operation time, and deceased number of retrieved lymph nodes. Low BMI group was associated with decreased rate of postoperative fever and decreased survival of patients.

The association between BMI and tumor stage was inconsistent in previous reports. Kim et al. reported that low BMI was associated with more advanced tumor stage [4]. Chen et al. reported that low BMI was associated with increased rate of lymph node metastasis and advanced tumor stage, but not associated with tumor depth [11]. However, no association between BMI and tumor stage has also been reported [12, 17]. The inconsistent results may attribute to many reasons, such as inclusion and exclusion criteria, sample size, cut off value of BMI, race, etc. As gastrointestinal malignancy, gastric cancer always accompanied with severe weight loss and cachexia [18]. Thus, advanced gastric cancer may be more inclined to exist in patients with low BMI. In our present study, we also found that BMI was inversely associated with tumor depth, lymph node metastasis and tumor stage.

The impact of BMI on intraoperative conditions were also widely investigated. Patients with high BMI was reported to be associated with increased blood loss [19, 20], increased operation time [17, 21] and decreased number of retrieved lymph nodes [10, 22] in most of previous reports. However, no association between BMI and intraoperative conditions has also been reported [23, 24] occasionally. Based on clinical experiences, obesity was thought to be associated with thick abdominal wall and massive adipose tissue in the abdomen, which will increase the difficulty of surgical resection [17]. Thus, the blood loss was increased and operation time was prolonged [11]. Furthermore, lymph nodes located deep in adipose tissue around major vessels were always difficult to remove in high BMI patients [10]. In our present study, we also found that high BMI was also associated with increased blood loss, operation time and decreased number of retrieved lymph nodes. No difference was found between low and normal BMI patients. The results were consistent with most of the previous reports.

From the surgical point of view, high BMI patients was thought to be associated with increased postoperative complications due to the prolonged operation time and increased blood loss. This has been confirmed by most of the previous reports. Kulig et al. reported that higher BMI was associated with higher rates of intra-abdominal abscess and cardiopulmonary complications [22]. Kim et al. also reported that obese was associated with higher rate of intra-abdominal abscess, wound problems and overall complications [20]. Hirao et al. showed that overweight was an independent risk factor for surgical site infection [19]. This risk may attribute to greater wound size and decreased oxygen tension in relatively avascular adipose tissue in overweight patients [19]. In addition, high BMI was also reported to be associated with anastomotic leak [10]. Theoretically, massive abdominal adipose tissue would result in a thick mesentery and increased tension on an anastomosis, which may result in anastomotic leakage [10]. However, no association between BMI and postoperative complications was also reported [12, 17]. In our present study, we found that normal and high BMI group was associated with increased rate of postoperative fever. The inconsistence of the results may attribute to sample size, type of gastrectomy, surgical techniques and perioperative nursing and treatment.

It was well known that overweight and obesity was a risk factor of death in general population [25]. However, “obesity paradox” has been proposed recently, referring to better prognosis of mildly obese patients after surgery [11]. The association between BMI and prognosis of patients after radical gastrectomy has also been widely investigated. Chen et al. reported that BMI was inversely associated with the prognosis of patients [11]. Tokunaga et al. reported that overweight patients had better prognosis after gastrectomy [26]. However, also with relatively large sample size, no association was found between BMI and prognosis of gastric cancer [10, 22, 27]. In our present study, low BMI was associated with decreased prognosis of gastric cancer. However, the prognosis was comparable between patients with normal and high BMI. It was reported that gastrectomy may result in 5%–19% body weight loss [26]. Thus, overweight patients may achieve ideal body weight years after gastrectomy, which may result in better prognosis. It was reported that cancer patients with low BMI was always accompanied by low hemoglobin and albumin levels which may due to poor nutritional status and cachexia [11]. The malnutrition in turn will impair the anti-tumor immunity of patients [28]. In the subgroup analysis in our present study, only the prognosis of patients with stage III disease was significantly influenced by BMI, which indicated that patients with normal and high BMI might be more able to bear cancer related malnutrition and stress.

There were several limitations in our present study. First, it was a single center’s experience with limited sample size, which may result in bias during analysis. Multi-center study with larger sample size was needed to confirm our results. Second, we only analyzed the impact of BMI at diagnosis on the clinical outcomes of patients. The impact of body weight loss before surgery on the clinical outcomes of patients were not analyzed. Third, as there were only twenty-three obese patients (BMI ≥ 30 Kg/m^2^) in our present study. We only divided patients into low, normal and high BMI groups. The impact of obesity on the clinical outcomes of gastric cancer was not independently analyzed. Fourth, it was reported that visceral fat area may be superior to BMI to predict the risk of gastrectomy. With regret, visceral fat area of patients was not evaluated in our present study.

## Conclusions

BMI was inversely associated with tumor size, tumor depth, LNM and tumor stage. High BMI was associated with increased blood loss and operation time, and deceased number of retrieved lymph nodes. Low BMI was associated with decreased rate of postoperative fever and decreased survival of patients.

## Abbreviations

BMI: body mass index
LNM: lymph node metastasis
